# Supplementary material for: Pirfenidone Inhibits Alveolar Bone Loss in Ligature-Induced Periodontitis by Suppressing the NF-κB Signaling Pathway in Mice
Source: Int J Mol Sci. 2023 May 12;24(10):8682. doi: 10.3390/ijms24108682 (PMC10218211; doi:10.3390/ijms24108682)
Supplement: Supplementary file 1 [file ijms-24-08682-s001.zip › ijms-2337813-supplementary.pdf]

International Journal of *Molecular Sciences*

MDPI

Supplementary Materials

Pirfenidone Inhibits Alveolar Bone Loss in Ligature-Induced Periodontitis by Suppressing the NF-κB Signaling Pathway in Mice

Zijiao Zhang [1,2], Juhan Song [1,2], Seung-Hee Kwon [1,2], Zhao Wang [1,2], Suk-Gyun Park [1,2], Xianyu Piao [1,2], Je-Hwang Ryu [1,2], Nacksung Kim [2,3], Ok-Su Kim [2,4], Sun-Hun Kim [2,5] and Jeong-Tae Koh [1,2,*]

1 Department of Pharmacology and Dental Therapeutics, School of Dentistry, Chonnam National University, Gwangju 61186, Republic of Korea
2 Hard-Tissue Biointerface Research Center, School of Dentistry, Chonnam National University, Gwangju 61186, Republic of Korea
3 Department of Pharmacology, Chonnam National University Medical School, Gwangju 61469, Republic of Korea
4 Department of Periodontology, School of Dentistry, Chonnam National University, Gwangju 61186, Republic of Korea
5 Department of Oral Anatomy, School of Dentistry, Chonnam National University, Gwangju 61186, Republic of Korea
* Correspondence: jtkoh@chonnam.ac.kr

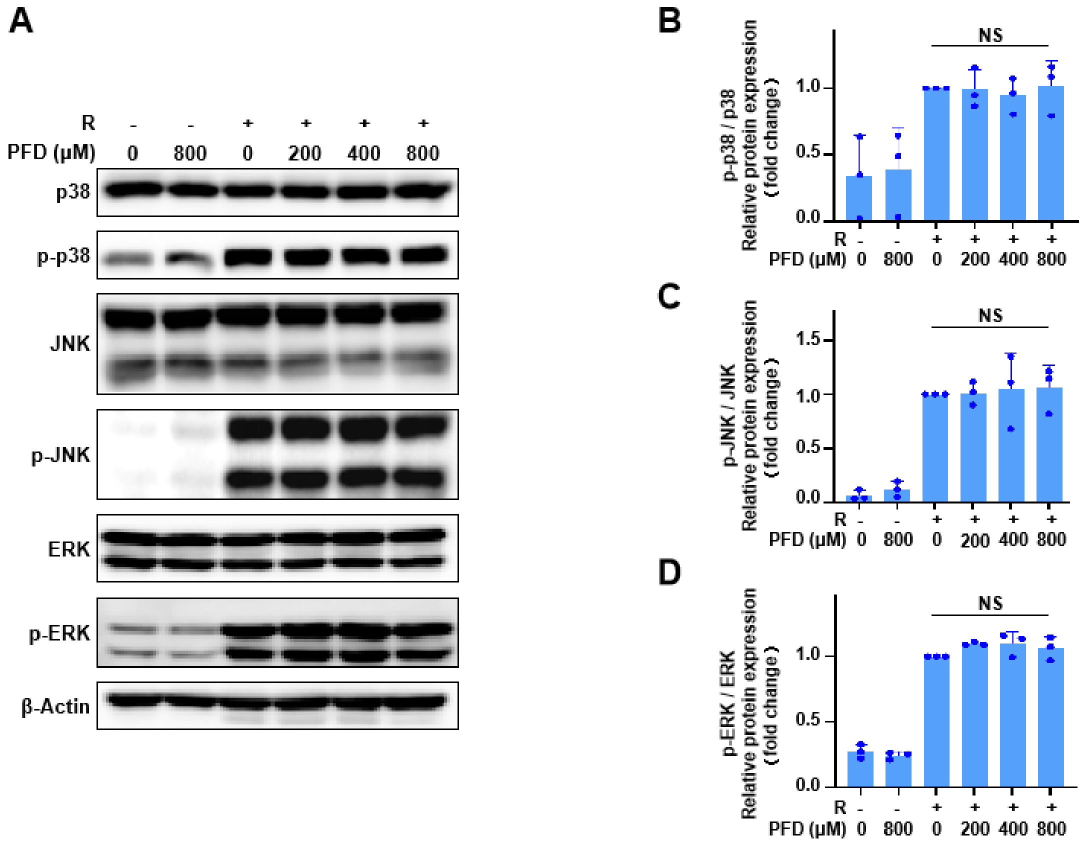

Figure S1. PFD has no effect on RANKL-induced MAPK pathway in BMMs. BMMs were cultured with or without PFD in GM containing M-CSF (30 ng/ml) and RANKL (100ng/ml) for 15 minutes. (**A**) Western blot analysis. (**B-D**) Quantitative densitometric analysis of (A). Pirfenidone, PFD. R, RANKL. All data represent the mean ± SD. NS, non-significant compared with the RANKL group. n = 3.

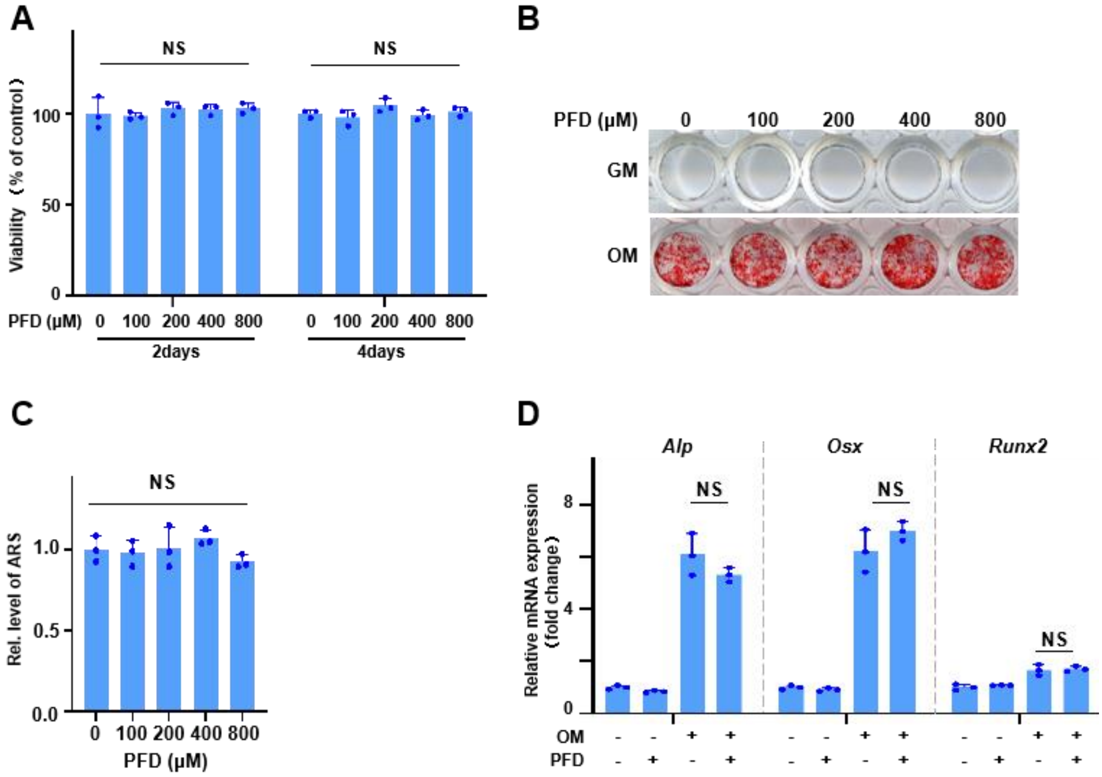

Figure S2. PFD has no effect on BMP2-induced osteoblast differentiation in BMSCs. (**A**) MTT assay. (**B**) Alizarin red stain (14 days). (**C**) The quantitative analysis of ARS staining (B). (**D**) Real-time PCR (10 days). Pirfenidone, PFD. GM, growth medium; OM, osteogenic medium. All data represent the mean ± SD. NS, non-significant. n = 3.
